# Ocular infrared thermography combined with stacked ensemble machine learning enables contact-free rectal temperature prediction in Murrah buffaloes

**DOI:** 10.3389/fvets.2026.1859307

**Published:** 2026-07-28

**Authors:** Ekta Hooda, Ashok Kumar Balhara, Gurpreet Kaur, Ashok Kumar Boora, S. K. Phulia, Mehar Singh Khatkar, Sunesh Balhara

**Affiliations:** 1 ICAR-Central Institute for Research on Buffaloes, Hisar, India; 2 Davies Livestock Research Centre, SAVS, The University of Adelaide, Roseworthy, SA, Australia

**Keywords:** ablation study, clinical utility, infrared thermography, medial canthus, Murrah buffalo, precision livestock farming, rectal temperature, stacked ensemble learning

## Abstract

**Introduction:**

Accurate core body temperature monitoring is central to health assessment in buffaloes, yet conventional rectal thermometry demands physical restraint and direct animal contact, causing stress to animals and posing injury risk to handlers.

**Methods:**

This study developed and validated a contact-free prediction framework combining ocular infrared thermography with stacked ensemble machine learning, using medial canthus temperatures of both eyes alongside environmental variables as predictors of rectal temperature in 471 adult female Murrah buffaloes. Normalisation parameters were derived solely from the training set. Eight supervised algorithms were trained under five-fold cross-validation; the five best-performing learners were integrated into a stacked ensemble with a penalised linear meta-learner. A 48-h continuous monitoring experiment (*n* = 10) further evaluated time-series concordance.

**Results:**

On the independent test set, the stacked ensemble achieved the strongest performance (RMSE = 0.2089, 95% CI: 0.179–0.240; *R*^2^ = 0.6152, 95% CI: 0.467–0.718), with random forest the best single learner. Clinically, 96.8% of predictions fell within ±0.5 °C of measured rectal temperature. Ablation analysis showed left eye temperature alone explained 36.6% of variance, rising to 56.0% with environmental variables and 61.5% with the full feature set. In continuous monitoring, LT, RT, and time were significant predictors (marginal *R*^2^ = 0.651; conditional *R*^2^ = 0.763), and Bland–Altman analysis confirmed a consistent mean bias of 1.5 °C between rectal and left eye readings.

**Discussion:**

Ocular medial canthus thermography combined with ensemble machine learning offers a reliable, welfare-friendly alternative to rectal thermometry, with practical potential for integration into precision livestock health monitoring systems.

## 1 Introduction

Core body temperature monitoring of animals is essential for diagnosing their physiological state and assessing their health and well-being. While rectal thermometry has long been considered the most precise method, its invasive nature creates practical difficulties, including contamination due to fecal matter, stress for the animal, and handling challenges (1, 2). It is especially challenging to handle buffaloes when their rectal temperatures are taken. Due to their large body size and reactivity to handling, they often require physical restraint during routine rectal thermometry, which increases the danger of injury to the handlers as well as animals. The challenges of rectal thermometry highlight the value of developing safer, labor-saving solutions, such as automated and remotely operated tools for buffalo temperature monitoring.

Alternatives include wearable sensors, such as rumen bolus sensors, which monitor rumen temperature continuously and have been shown to be a proxy for core body temperature (3). Another alternative is the use of infrared thermography technology (IRT), a non-invasive, non-contact technique that records infrared radiation emitted from the body surface to assess surface temperature distribution (4), which may be influence by underlying blood flow and other physiological processes (4). The output is obtained via a thermogram used to estimate the body surface temperature. In the veterinary sciences, IRT has been used previously to study mastitis, lameness, and various respiratory diseases (5–7). The eyes have been shown to be closely linked with core body temperature due to the presence of abundant blood vessels in the region (8). This association has been demonstrated in cows and ponies (9, 10) and rectal temperature has been predicted via the IRT-derived eye temperature with the help of the stepwise multiple regression (MLR) model (11).

Machine learning models are increasingly used in veterinary sciences for prediction and decision support tasks including the estimation of physiological parameters from thermographic data (12–14). Different machine learning algorithms capture different aspects of dataset. Random forest (RF) and LightGBM are well suited for large datasets and often deliver strong accuracy (15, 16). Gradient boosting (GB) and XGBoost are effective at reducing bias and variance, whereas support vector machines (SVMs) perform reliably in high-dimensional spaces (17).

In the case of small datasets, *k*-nearest neighbours (*k*-NN) offers a simple but competent approach, relying on local pattern recognition to generate predictions (18). LASSO and ridge regression continue to be important tools for managing problems of overfitting and multicollinearity (19, 20). Ensemble learning (EL) links these individual models together and further improves their predictive ability. EL creates a more reliable and accurate prediction system by integrating these weak learners (or base learners) (21). Errors from one model are offset by those from the other models, which in turn leads to a better combined performance than that of individual models (22). EL also helps reduce the chances of overfitting via either averaging predictions, such as bagging, or by concentrating on difficult cases, such as in boosting (23). These models have shown great value in the field of animal health monitoring because they support better decision-making and protective strategies for veterinarians and farmers. Recently, EL combined with advanced computer vision techniques, such as convolutional neural networks (CNNs) and YOLO, has been used for cattle breed identification (24).

The objective of this study was to develop a non-invasive method for predicting rectal temperature in Murrah buffaloes using machine learning models that combine ocular infrared measurements at the medial canthus with environmental variables, including air temperature, background thermal radiation (measured as reflected temperature), and relative humidity. We hypothesized that bilateral medial canthus temperatures along with air temperature, relative humidity and reflected temperature would provide accurate, contact-free predictions of rectal temperatures, suitable for routine on-farm use.

## 2 Materials and methods

### 2.1 Experimental setup, ethical considerations and data collection

The study was carried out at the Indian Council of Agricultural Research (ICAR)—Central Institute for Research on Buffaloes in Hisar, Haryana, India from July 2023 to October 2024. A total of 471 adult female buffaloes were housed in an experimental shed to minimize direct sunlight during image acquisition. No animals were restrained specifically for the purpose of this experiment. The visible neck tether is part of the routine farm management and is normally used during milking. Ethical approval was obtained from the Institute Animal Ethics Committee (IAEC) under the letter IAEC-CIRB/2021-22/A/07, dated August 28, 2023.

**Figure 1:**
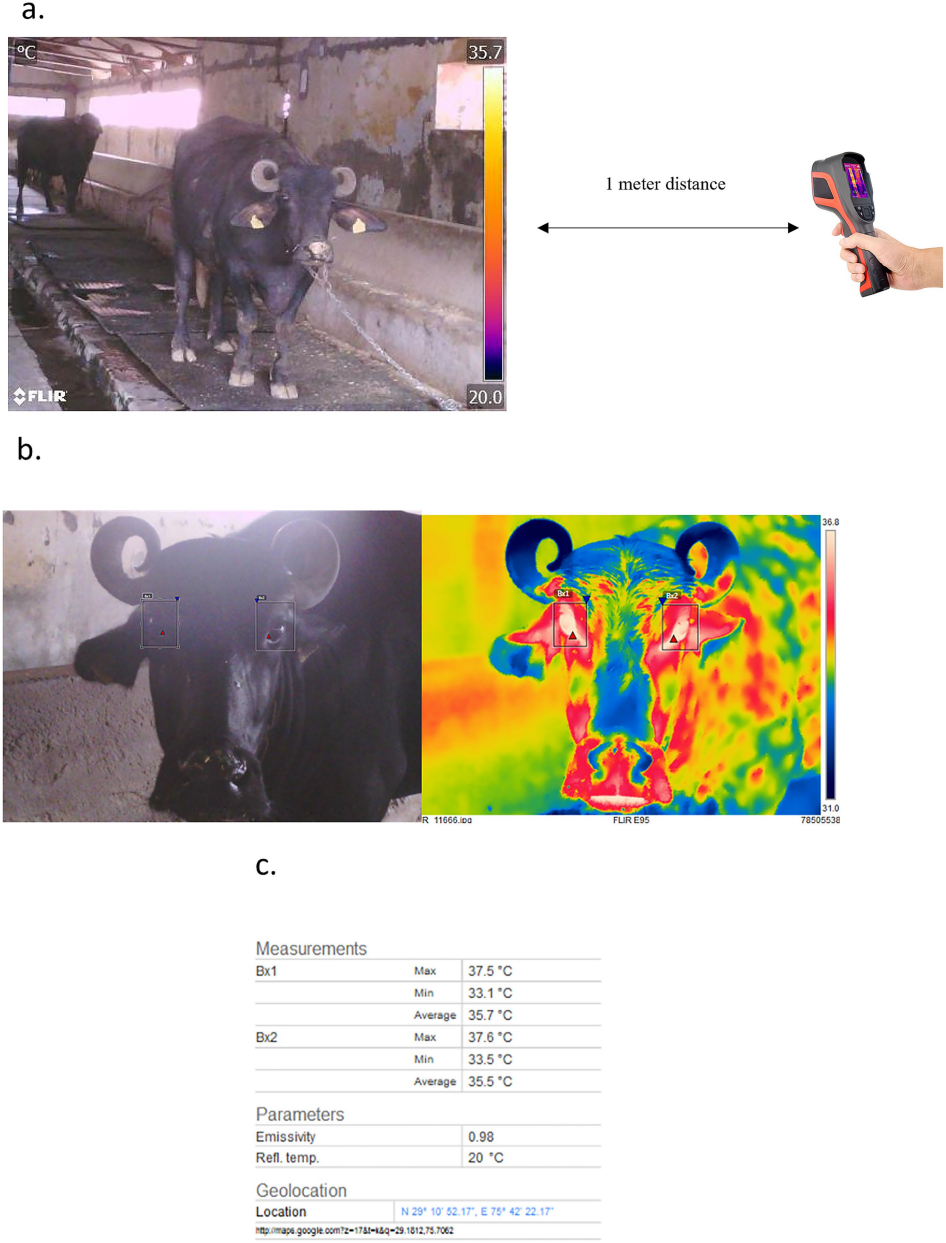
**(a)** Infrared thermographic setup in a controlled housing environment, capturing thermal images with a handheld thermal camera from 1 m. The neck tether visible in the image is the routine rope used during milking management at the Institute farm and was not an additional restraint applied for the purpose of the study. **(b)** A digital image and thermographic image of the buffalo’s head, capturing both eyes in one frame, focusing on medial canthus as the region of interest (ROI). **(c)** Tabulated thermal data for two anatomical regions (Bx1 and Bx2), showing maximum, minimum, and average temperatures, along with calibration parameters (emissivity of 0.98, reflected temperature and geolocation).

All 471 buffaloes included in the study were aged 4–8 years and had body condition scores ranging from 3.0–3.5 on a 5-point scale (25). All the animals were in mid- to late-lactation at the time of measurement, with no signs of systemic disease or ocular abnormalities. Infrared thermal images were captured via a handheld thermal camera (FLIR E95, USA) with a high infrared resolution of 464 × 348 pixels and an emissivity setting of 0.98, captured from a distance of 1 m (Figure 1). This working distance falls within published veterinary guidelines and has previously been shown to provide accurate and repeatable surface temperature measurements in large animals while maintaining a non-contact, welfare-friendly imaging setup (26, 27).

All imaging was performed in the afternoon (between 1,300 and 1,500 h) before milking for consistency and were distributed across all seasons within the study window (summer, monsoon, winter). Every clinically healthy, adult female Murrah buffalo presenting at the routine afternoon milking line during the study period was included. Buffaloes were standing in the routine head-bail stanchion with neck ropes used for daily feeding and milking. In order to minimise startling, the handler approached each animal slowly from the side and front. The thermal image of the head was acquired first while the animal stood quietly and the rectal temperature was then measured immediately. To ensure accurate thermal readings, the surrounding air temperature and relative humidity of the animals were recorded via a portable data logger (HTC-1, ACE Technologies, Korea). Rectal temperatures, measured via digital rectal thermometers (Omron Glass Mc 246 Digital Thermometer), were treated as the reference standard (ground truth) for model development.

### 2.2 Continuous monitoring experiment

An additional 48-h continuous monitoring experiment (in February 2025) was conducted to assess whether the time-series trend of rectal temperature measured by rectal thermometer can be tracked by time-series trend of medial canthus eye temperatures (left eye temperature and right eye temperature), extracted from thermograms under repeated-measures conditions. Ten clinically healthy adult Murrah buffaloes (2.5–3.5 years), not included in the 471 animal primary dataset were monitored under standard conditions, i.e., loose housing with partially covered concrete pens, access to shaded resting areas, *ad libitum* access to fresh water and a standard farm ration consisting of concentrates (wheat, maize, wheat bran, mustard cake, vitamins and salt), wheat straw, and green fodder (sorghum). Paired thermal images and rectal temperatures were recorded simultaneously every 4 h, giving 13 paired observations per animal at *t* = 0, 4, 8, 12, 16, 20, 24, 28, 32, 36, 40, 44 and 48 h, starting at 0900 h on day 1. Air temperature (AT), relative humidity (RH) and reflected temperature (RefT) were logged at every time point. Reflected temperature represents the radiation emitted by the surroundings that is reflected off the object into the camera. It must be supplied as a calibration input, along with emissivity, so that this reflected component can be subtracted from the detected signal when converting infrared radiation into surface temperature (4). The restraint conditions and the order of measurements were identical to those described in Section 2.1.

### 2.3 Data preprocessing, model development and configuration

The predicted (outcome) variable was the rectal temperature (RecT). The predictor variables are LT, RT, AT, RH and RefT. The dataset was pre-processed using FLIR Tools (version 6.4.18039.1003). For each animal, one frontal image was selected in accordance with the predefined inclusion criteria. Images were required to show the head in a straight frontal orientation, with both eyes fully visible and unobstructed, and to be in sharp focus without motion blur or artifacts within the region of interest (ROI). Thermograms were excluded if they exhibited poor quality, blurriness, artifacts, or head rotation/tilt that caused partial loss or distortion of either eye. The remaining thermal images (rainbow HC color palette, histogram equalized) were calibrated for air temperature and relative humidity. The medial canthus of the eye served as the ROI and was manually verified via the digital camera images.

For each retained thermogram, the medial canthus of the left and right eye was defined as the ROI. For each ROI, a small rectangular polygon was drawn manually in FLIR Tools, so as to enclose only the medial canthus area. Within each ROI, the maximum pixel temperature was extracted and used as eye temperature value (LT, RT) in all subsequent analysis. LT, RT, AT, RefT, RH (%) and rectal temperature for each animal were compiled into a dataset of 471 paired observations. Sixteen missing values were imputed via feature-wise means. The variables were standardized using *z*-score normalization (mean = 0, SD = 1) to correct for scale differences and to facilitate model convergence.

**Figure 2:**
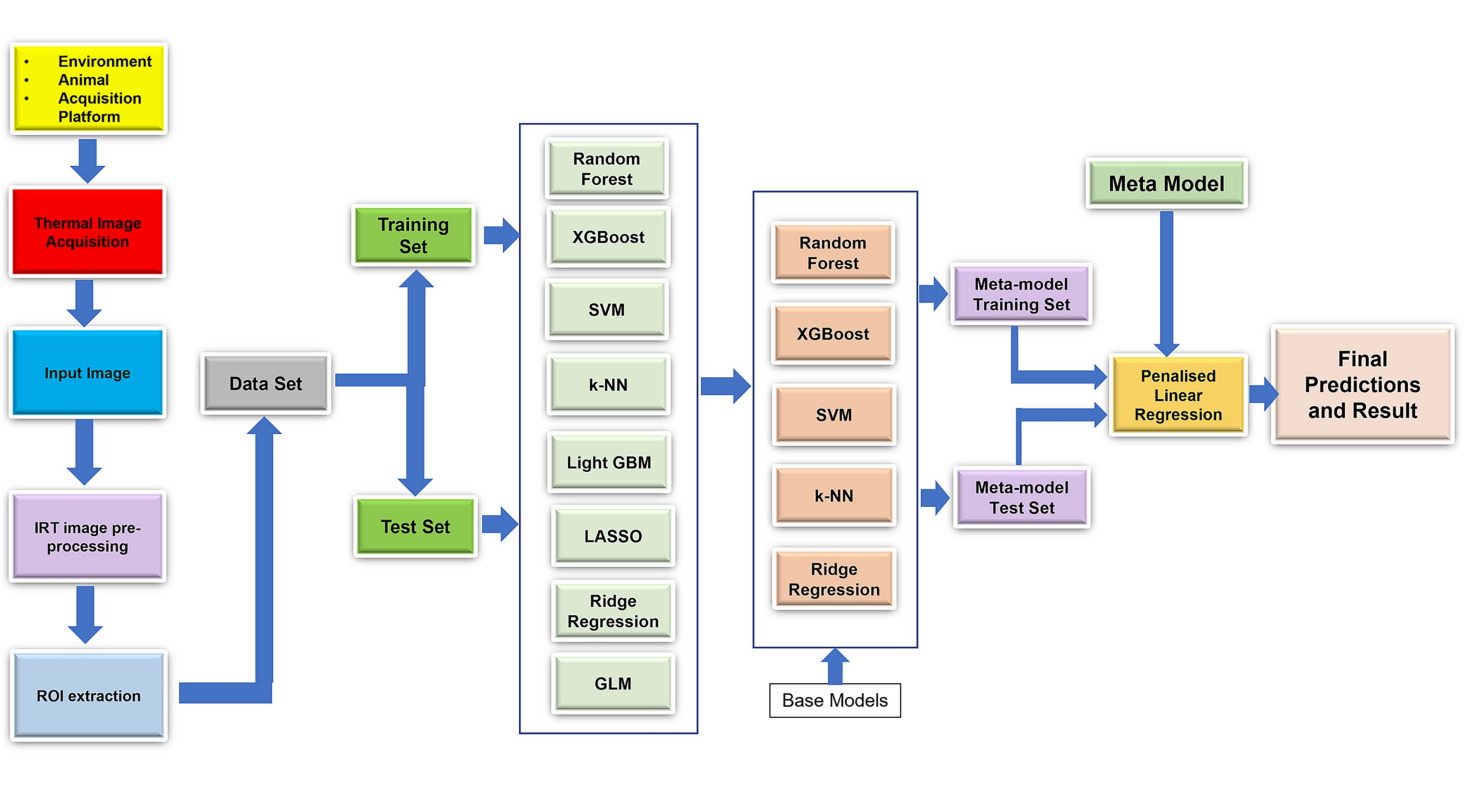
Rectal body temperature prediction framework’s technical roadmap describing various steps.

The statistical analysis was performed via RStudio (version 2024.04.2) using *caretEnsemble, caret, randomForest, xgboost, glmnet, kknn, lightgbm*, *e1071, lme4,* and *lmerTest* for model development, with *GGally, lattice* and *ggplot2* for visualization. Multiple statistical and ML models have been developed to predict rectal temperature *via* supervised ML algorithms (LGBM, RF, LASSO, SVM, *k*-NN, ridge regression, GLM and XGBoost) using the medial canthus temperature of the left and right eyes, air temperature, and relative humidity as predictors. These algorithms were selected *a priori* to represent complementary modeling paradigms: GLM and ridge regression as linear baselines; LASSO as a sparse penalized linear model; *k*-NN as a non-parametric instance-based learner; SVM (radial kernel) as a margin-based non-linear method; and RF, XGBoost and LGBM as tree-based ensemble methods capable of capturing higher-order interactions and non-linear relationships in tabular data. Hyperparameter optimization was implemented via a grid search to refine model performance. The best models, selected on the basis of error minimization (RMSE, MAE, MSE), were integrated into a stacked generalization ensemble. For stacked generalization, we defined a priori a heterogeneous set of base learners comprising RF, XGBoost, SVM, *k*-NN and ridge regression, with a penalized linear model fitted via the glmnet algorithm as the meta-learner. This configuration was chosen to maximize diversity in modeling assumptions (tree-based ensembles, margin-based, distance-based and penalized linear models) while avoiding redundancy among closely related algorithms; LGBM and LASSO were treated as alternative implementations of gradient boosting and penalized linear regression, respectively, and therefore were not included as additional base learners in the stack. Correlations among the prediction outputs of the individual models were examined to verify that the selected learners exhibited the non-identical prediction behavior required for stacked ensemble modeling. A pictorial representation of the process followed is shown in Figure 2.

An ablation study was performed to quantify the contribution of each feature group. Four configurations were tested using both RF and the stacked ensemble: LT alone; LT combined with environmental variables (AT, RefT, RH); both eye temperatures without environmental inputs; and complete feature set. Permutation-based feature importance was computed on the independent test set providing a model-agnostic measure that avoids training set bias.

### 2.4 Model validation and assessment

Out of a total of 471 records, 95 records (approximately 20%) were randomly selected and set aside as an independent test set. The remaining 376 records were used for model development through internal cross-validation. For the individual machine learning models and the base learners in the stacked ensemble, model development and hyperparameter tuning were performed using 5-fold cross-validation. The training dataset (*n* = 376) was partitioned into five approximately equal subsets, iteratively training the model on four subsets while validating it on the fifth subset. Each fold served as the validation set exactly once, ensuring a comprehensive performance evaluation across all the data points.

The final model’s accuracy was then measured on the independent test set (*n* = 95) that had been withheld from the entire development process. For the test set evaluation, model performance was evaluated using root mean squared error (RMSE), mean squared error (MSE), mean absolute error (MAE), *R*^2^ and adjusted *R*^2^ (reported in Table 1). To account for estimation uncertainty due to limited sample size, 95% Bootstrap confidence intervals (1,000 resamples, percentile method) were calculated for RMSE and *R*^2^. Clinical utility was further assessed by determining the proportion of predictions within ±0.5 °C of the rectal reference temperature, a tolerance widely accepted in veterinary thermometry. The results were presented as scatterplots of the predicted temperature versus actual observations, along with correlation coefficients, to assess the strength of the relationship between the predicted and observed values.

**Table 1:** Evaluation metrics of different machine learning models for the prediction of rectal temperature in buffaloes on the basis of predictions on the independent test set with 95% bootstrap confidence intervals (1,000 resamples, percentile method).

Model	RMSE (95% CI)	MSE	MAE (95% CI)	*R*^2^ (95% CI)	Adj. *R*^2^
LightGBM	0.2142 [0.1856, 0.2422]	0.0459	0.1748 [0.1515, 0.1991]	0.5954 [0.4608, 0.6942]	0.5726
XGBoost	0.2102 [0.1794, 0.2431]	0.0441	0.1674 [0.1433, 0.1919]	0.6106 [0.4528, 0.7150]	0.5887
Random forest	0.2101 [0.1797, 0.2418]	0.0442	0.1694 [0.1460, 0.1933]	0.6103 [0.4501, 0.7151]	0.5884
LASSO	0.2576 [0.2259, 0.2878]	0.0664	0.2084 [0.1793, 0.2377]	0.4145 [0.2213, 0.5521]	0.3816
Ridge	0.2542 [0.2232, 0.2829]	0.0646	0.2061 [0.1779, 0.2352]	0.4299 [0.2358, 0.5630]	0.3978
*k*-NN	0.2247 [0.1939, 0.2569]	0.0505	0.183 [0.1583, 0.2089]	0.5544 [0.4081, 0.6611]	0.5294
SVM	0.2276 [0.1867, 0.2648]	0.0518	0.1777 [0.1513, 0.2072]	0.5429 [0.3628, 0.6797]	0.5172
GLM	0.2567 [0.2255, 0.2862]	0.0659	0.208 [0.1784, 0.2381]	0.4188 [0.2077, 0.5588]	0.3861
Stacked ensemble	0.2089 [0.1786, 0.2402]	0.0436	0.1679 [0.1446, 0.1915]	0.6152 [0.4670, 0.7176]	0.5935

RMSE, root mean squared error; MSE, mean squared error; MAE, mean absolute error; *R*^2^, coefficient of determination.

### 2.5 Modeling and analysis of rectal-IR thermal temperatures in continuous monitoring

For the analysis of the data generated from this experiment, descriptive statistics and Pearson’s correlations were calculated for the rectal, thermal, and micro-environmental variables. Bland–Altman analysis and a linear mixed-effects model (LMM) using restricted maximum likelihood (REML) with Satterthwaite’s method were used to evaluate the effectiveness of medial canthus temperature (LT and RT), extracted from IRT as the predictor in predicting the rectal temperature of the animals. The model was established as follows:


$$\text{RecT}=\beta_{0}+\beta_{1}\mathrm{LT}+\beta_{2}\mathrm{RT}+\beta_{3}\mathrm{AT}+\beta_{4}\mathrm{RH}+\beta_{5}\text{Time}+u_{\text{Animal}\_\mathrm{ID}}+\varepsilon$$

The model included fixed effects for LT, RT, AT, RH and time, with animal ID as a random effect. The model fit was evaluated via the AIC/BIC and the marginal and conditional *R*^2^ values.

This dataset was also used to evaluate the performance of the ensemble model developed for predicting rectal body temperature in a continuous monitoring scenario.

## 3 Results

### 3.1 Descriptive summary

The mean temperatures in the medial canthus region for the left and right eyes were 37.08 ± 0.50 °C and 37.11 ± 0.46 °C, respectively, and the rectal temperature was 38.26 ± 0.37 °C. The average eye temperature (maximum for ROI) (37.10 °C ± 0.46 °C) and rectal temperature were significantly correlated (Pearson correlation coefficient *r* = 0.70).

**Figure 3:**
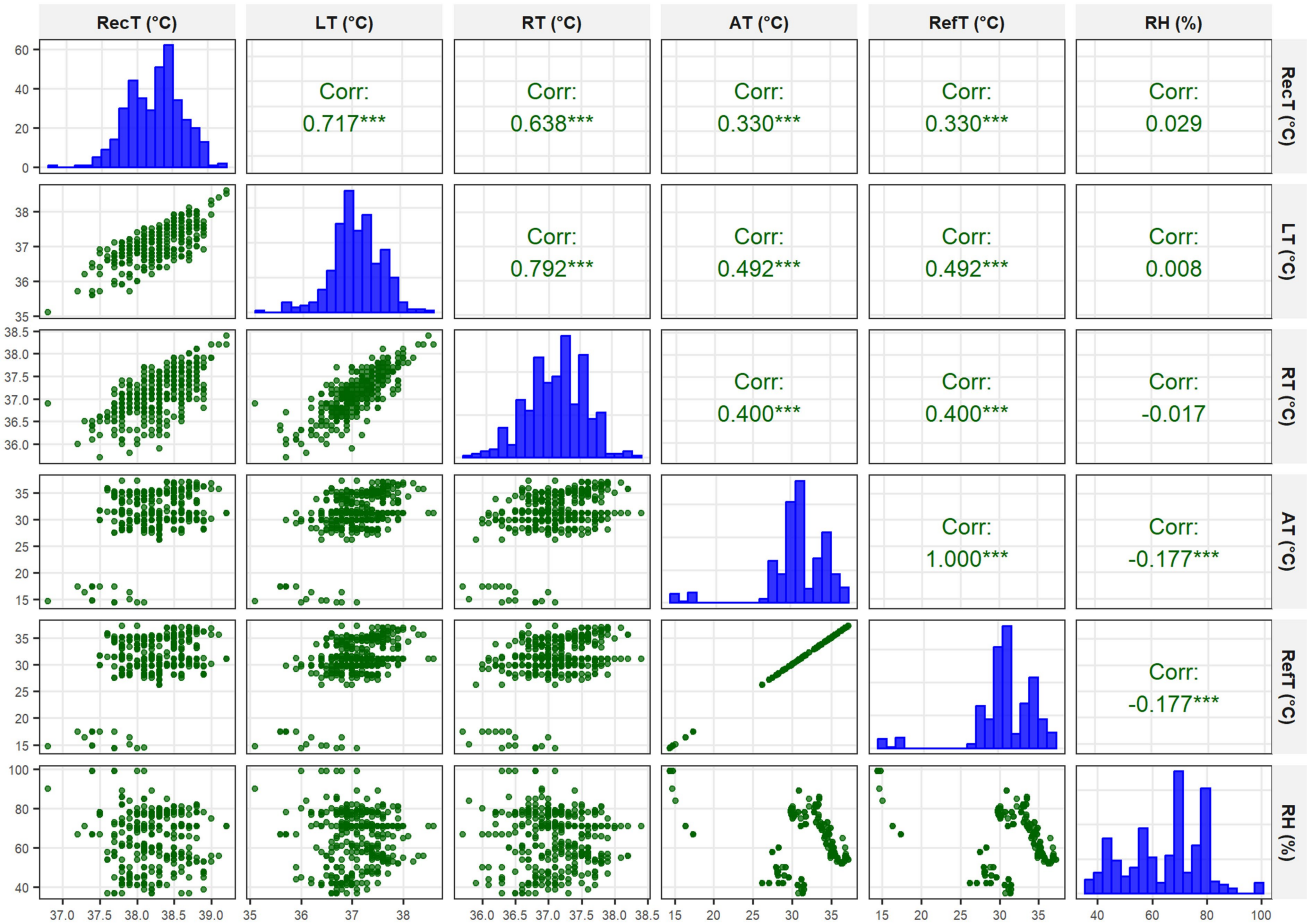
Correlation matrix heatmap of temperature and environmental variables (RecT, rectal temperature; LT, left eye temperature; RT, right eye temperature; AT, air temperature; RefT, reflected temperature; RH, relative humidity).

Figure 3 displays a pair plot featuring correlation coefficients and histograms, which illustrates the relationships among the measured variables, namely, the rectal temperature (RecT), left eye (LT), right eye temperature (RT), air temperature (AT), reflected temperature (RefT) and relative humidity (RH). The analysis revealed significant positive correlations between RecT and LT (*r* = 0.717, *p* < 0.001) as well as RT (*r* = 0.638, *p* < 0.001), suggesting their potential as important predictors of rectal temperature. A statistically significant positive correlation was also observed between LT and RT (*r* = 0.792), suggesting that either eye could be used independently in predictive modeling without much information loss. A moderate positive correlation was observed between RecT and AT (*r* = 0.330, *p* < 0.001), indicating that ambient conditions had a measurable but weaker association with rectal temperature than ocular thermal traits. The scatter plots showed clearer linear pattern trends for strongly correlated variables, whereas weaker associations displayed greater dispersion.

### 3.2 Feature screening and importance

**Figure 4:**
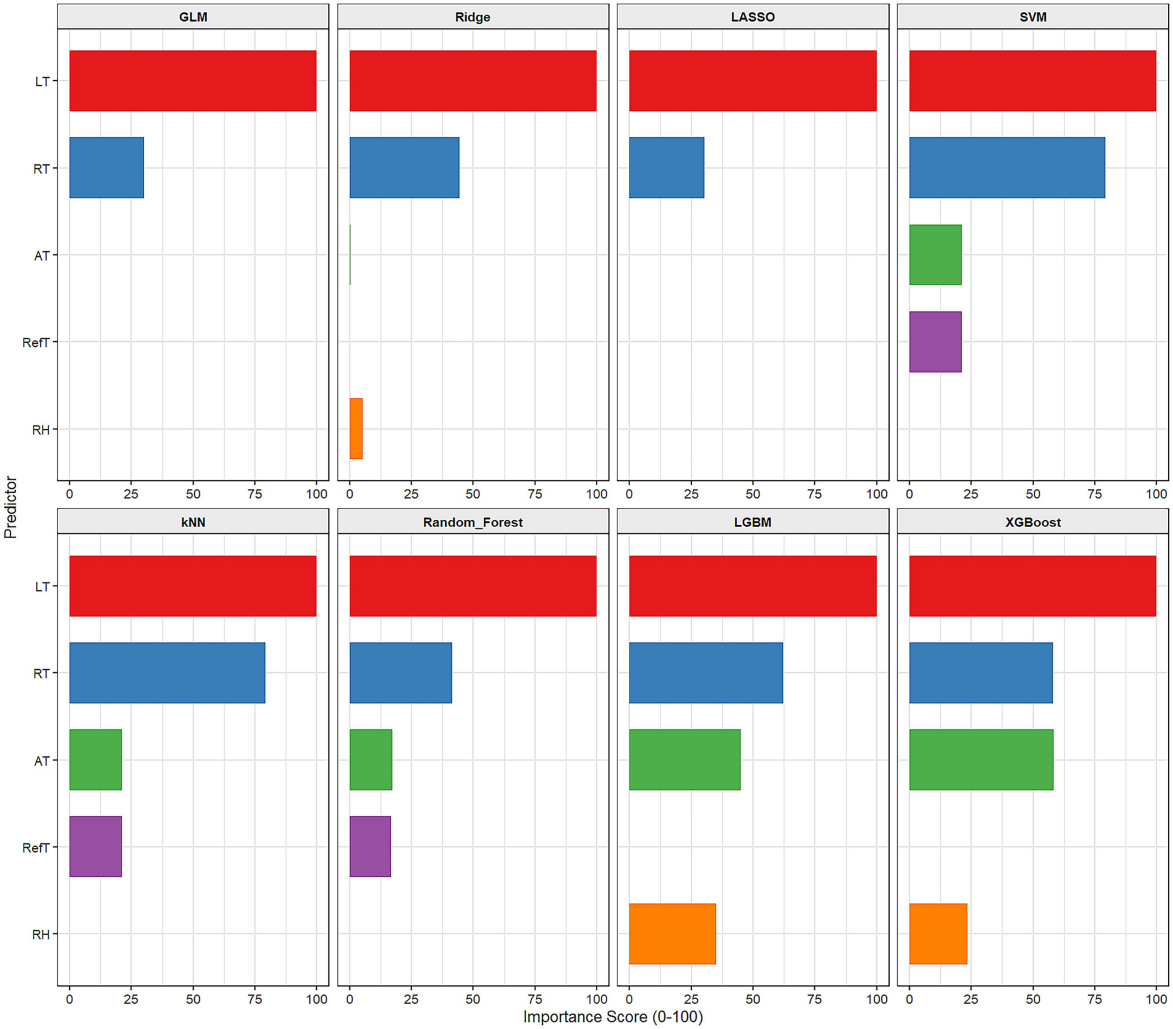
Permutation-based importance scores for the predictive features in each model. (LT, left eye temperature; RT, right eye temperature; AT, air temperature; RefT, reflected temperature; RH, relative humidity).

Feature importance by Permutation method was assessed for all models and temperature-related features, particularly LTs, consistently emerged as the most significant predictor across all the models, as illustrated in Figure 4. This highlights that left eye temperature is the most critical and comparatively superior predictor of rectal temperature in buffaloes. The importance values for RT, AT, RefT and RH were positive but consistently lower than that of LT across all models. This indicates that each of these predictors, taken individually, contributed less to model accuracy than LT did.

### 3.3 Base model selection

**Figure 5:**
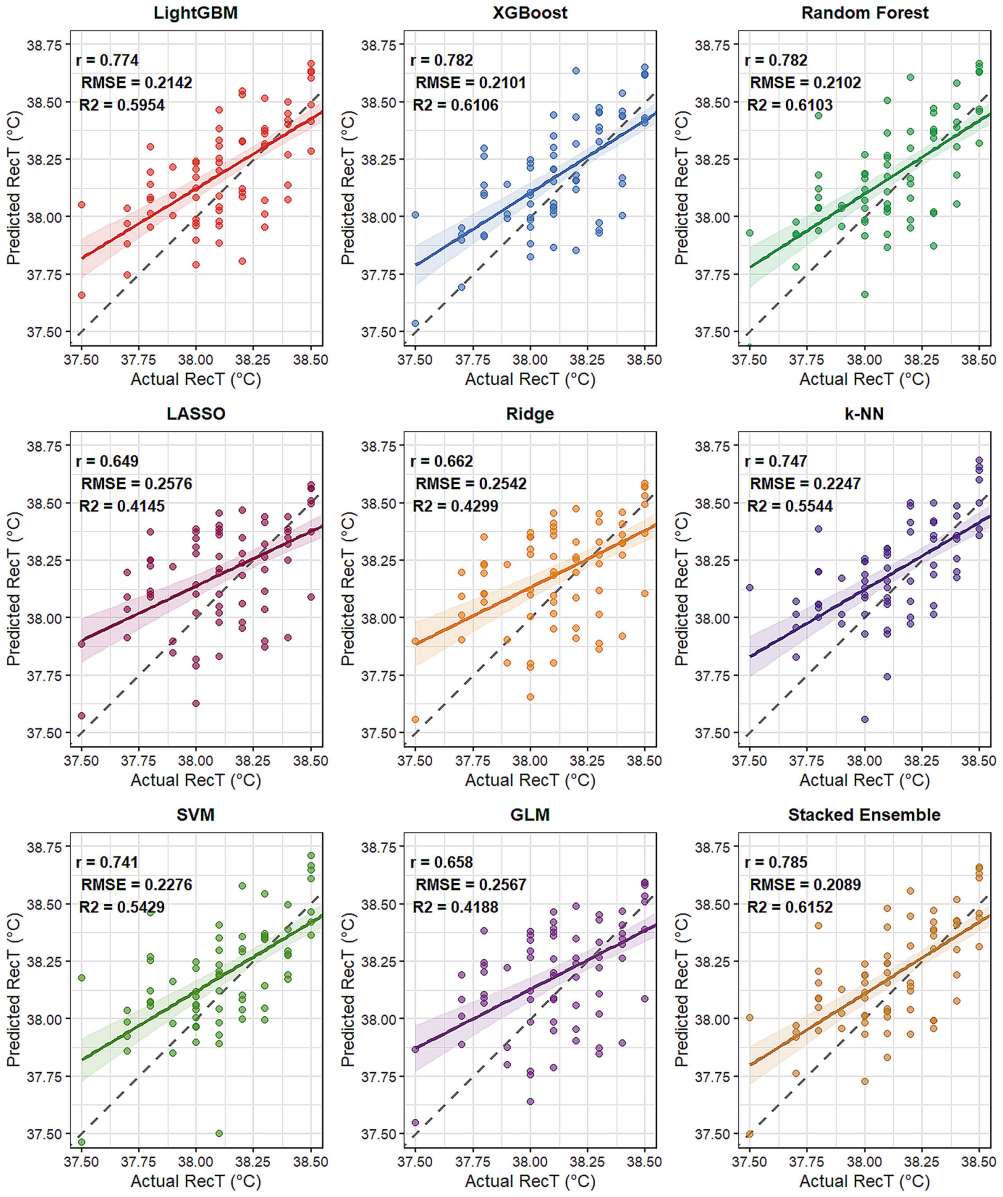
Actual vs. predicted (based on independent test data) plots for nine models under study (actual temperatures on x-axis and predicted temperatures on y-axis).

In the first phase of the study, eight ML models (LightGBM, XGBoost, RF, LASSO, ridge regression, *k*-NN, SVM, and GLM) were employed to predict the rectal temperature. Hyperparameter tuning was performed using Grid Search cross-validation to find the optimal settings (Table 2). On the basis of the internal evaluation, both the RF and the stacked ensemble demonstrated strong performance. For RF, the optimal configuration achieved a mean cross-validated RMSE of 0.2201 and a coefficient of determination (*R*^2^) of 0.6724. For the stacked ensemble, the penalized linear meta-learner (glmnet) combining RF, XGBoost, SVM, *k*-NN and ridge regression attained a similar cross-validated RMSE (0.2188) and slightly higher cross-validated *R*^2^ (0.6777), indicating good internal fit for both models. Performance evaluation metrics based on an independent test set for all the models were also computed (Table 1). On the independent test set, the stacked ensemble achieved the strongest overall performance (RMSE = 0.2089, 95% CI 0.179–0.240; *R*^2^ = 0.6152, 95% CI 0.467–0.718). The RF model was the best performing single learner (RMSE = 0.2102, *R*^2^ = 0.6103), only slightly behind the ensemble. Bootstrap CI confirmed that the advantage of tree-based methods over linear models (LASSO, Ridge, GLM) was consistent. Clinically, 96.8% of ensemble predictions fell within ±0.5 °C of the rectal reference temperature. A comparative analysis of model-predicted versus actual RecT values, along with correlation coefficients, is illustrated in Figure 5.

**Table 2:** Parameters used in various models under study.

Model	Parameters used
Light GBM	nrounds = 100, max_depth = 5, eta = 0.1, gamma = 0, colsample_bytree = 1.0, min_child_weight = 1, subsample = 1.0
XGBoost	nrounds = 100, max_depth = 7, eta = 0.1, gamma = 0, colsample_bytree = 0.8, min_child_weight = 1, subsample = 0.8.
Random forest	mtry = 2, trees = 500
LASSO	alpha = 1 (held constant) and lambda = 0.02
Ridge	alpha = 0 (held constant) and lambda = 0.02
*k*-NN	*k* = 7, weights = uniform
SVM	kernel = radial, cost = 2, kernel width *σ* ≈ 0.3711

A stacked ensemble learning model was therefore retained as a competitive alternative to RF, integrating base models with a meta-learner. Figure 5 shows a comparison between the actual and predicted values from the ensemble model, revealing a high correlation coefficient value of 0.785, effectively demonstrating the overall trend in the data.

Within the stacked ensemble, the meta-learner assigned the largest weight to RF (60% of the total absolute model weight), with XGBoost followed with 37%. The two are complementary, with RF reducing variance through bagging and XGBoost reducing bias through boosting. Ridge regression contributed a modest 3.4%. In contrast, the coefficients for SVM and *k*-NN were reduced to zero by the penalized meta-learner, indicating that these models did not provide additional predictive information beyond that already captured by the tree-based learners and ridge regression.

The residual analysis (Supplementary Figure S1) conducted for the stacked ensemble model showed a scatterplot of the residuals against the predicted temperatures, revealing random scatter around the middle (red dashed line), indicating no systematic deviations, overfitting or biased predictions. A histogram of residuals showed a near-normal distribution, bell-shaped distribution centered at zero, suggesting symmetric errors and reliable model performance. Additionally, calibration plots further confirmed that neither model exhibited systematic over- or under-prediction across the observed temperature range (Supplementary Figure S2).

### 3.4 Ablation study

An ablation study systematically evaluated the contribution of each feature group on the test set. Using LT alone resulted in substantially lower accuracy (*R*^2^ = 0.366, RMSE = 0.268; Stacked: *R*^2^ = 0.366) confirming that ocular temperature by itself is insufficient (Table 3). Adding environmental variables markedly improved performance (RF: *R*^2^ = 0.563, RMSE = 0.223; Stacked: *R*^2^ = 0.560). Including both eye temperatures without environmental inputs (LT + RT: *R*^2^ = 0.418) produced intermediate results. All five predictors together achieved the highest accuracy (stacked *R*^2^ = 0.615), demonstrating that bilateral ocular measurements combined with environmental calibration provide complementary, non-redundant information although LT with environment alone may also be sufficient.

**Table 3:** Results of ablation study with different feature sets.

Feature set	Number of features	RF RMSE	RF *R*^2^	Stacked model RMSE	Stacked model *R*^2^
LT only	1	0.2680	0.3662	0.268	0.3662
LT + environment	4	0.2225	0.5634	0.2235	0.5595
Both eyes (no environment)	2	0.2584	0.4108	0.2568	0.4184
All features (LT + RT + AT + RefT + RH)	5	0.2102	0.6103	0.2089	0.6152

### 3.5 Continuous monitoring experiment

**Figure 6:**
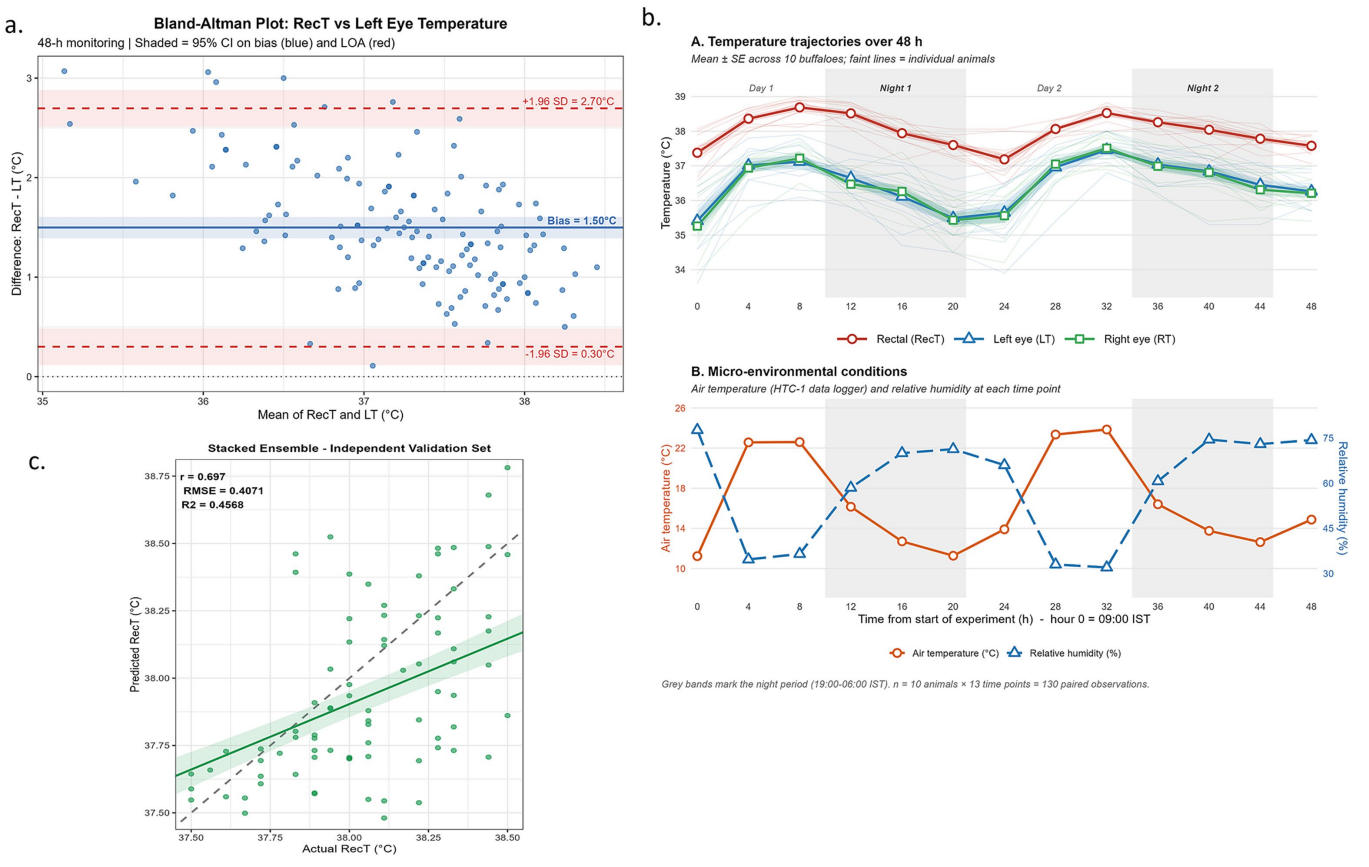
**(a)** Bland–Altman plot showing agreement between RecT and LT and displaying a mean difference of 1.5 °C between the two with a systematic bias. **(b)** Timeline trajectory for 48 h continuous monitoring experiment. **(c)** Plot for actual rectal temperatures vs. predicted rectal temperatures using ensemble model for 48 h continuous monitoring experiment.

During the 48-h monitoring period, the reflected temperature ranged from 11.23 °C to 23.85 °C, and the relative humidity ranged from 32 to 78% across the 4-h intervals. Descriptive statistics by time point revealed that the mean RecT ranged from 37.2 °C to 38.7 °C. The corresponding eye temperatures remained consistently lower than the rectal temperatures by approximately 1.5 °C at each time point (Figure 6b). Pearson’s correlation analysis again indicated significant positive correlations between RecT and LT (*r* = 0.736, *p* < 0.001), and between RecT and RT (*r* = 0.723, *p* < 0.001). Bland–Altman analysis showed a mean difference of approximately 1.5 °C indicating that, on average, the RecT is 1.5 °C higher than LT (Figure 6).

Repeated measurements were taken on each buffalo, and individual animals differed, so a linear mixed-effects model (LMM) was used to predict RecT. The results revealed that LT and RT are significant predictors (*p* < 0.001) of RecT, whereas the micro-environment did not notably influence the outcome in this experiment (Table 4). In particular, the time of recording (0–24 h) was the most significant predictor (*p* < 0.0001). The random intercept for animal ID had a variance of 0.04 indicating a modest variability among individual buffaloes. The marginal *R*^2^ (reflecting variance explained by fixed effects) was 0.651, whereas the conditional *R*^2^ (including random effects) rose to 0.763, indicating that incorporating individual differences in terms of random effects among the buffaloes significantly improved the model’s explanatory power. The residual diagnostics showed no major deviation from model assumptions, indicating an adequate fit to the data.

**Table 4:** Fixed effects estimated from the LMM-REML predicting rectal temperatures in buffaloes.

Predictor	Estimate	Standard error	*t*-value	*p*-value
(Intercept)	17.916501	1.990526	9.001	3.26e−15***
LT	0.28825	0.087713	3.286	0.001333**
RT	0.28841	0.095316	3.026	0.003023**
AT	−0.023209	0.032807	−0.707	0.480702
RH	−0.00611	0.008651	−0.706	0.481436
Time	−0.008975	0.002288	−3.922	0.000149***

** *p* < 0.01; *** *p* < 0.001.

To evaluate ensemble model’s predictive performance further, an additional validation dataset was utilized. In this study, thermally associated parameters, namely, LT and RT, were used as inputs to the ensemble model for the purpose of rectal temperature prediction. The analysis revealed a Pearson correlation coefficient of 0.697 between the measured and predicted temperatures, indicating strong predictive accuracy, which was lower than the accuracy observed in our primary dataset (Figure 6c).

## 4 Discussion

This study demonstrated that infrared thermal imaging of the ocular region, combined with ensemble machine learning can accurately predict rectal temperature in buffaloes with clinically acceptable precision; 96.8% of predictions fell within ± 0.5 °C of the rectal reference while entirely eliminating the need for physical restraint or animal contact. Multiple machine learning models have been developed and compared to predict rectal temperature in buffaloes using infrared thermography (IRT) of the eye region. The cross-validation performance was high for both random forest (RF) and the stacked ensemble (*R*^2^ ~ 0.67–0.68), with the ensemble showing a modest improvement. This trend persisted in the independent test set, where the stacked ensemble marginally surpassed RF, indicating consistently superior performance across both internal and external evaluations. Bootstrap confidence intervals confirmed that this advantage was consistent: the stacked ensemble’s *R*^2^ CI overlapped substantially with RF, indicating that the two models are statistically comparable at this sample size. The eye region of animals is a highly vascular area, with sparse fur coverage, making it a reliable site for non-invasive thermal measurements in cattle and ponies (9, 28–31).

Machine learning models using IRT derived temperatures have been applied in previous studies for lameness detection, thermal condition classification, and mastitis detection (32–34). This study extends the application of ensemble techniques to predict rectal temperature in buffaloes. The stacked ensemble learning model comprises multiple base learners, namely, RF, XGBoost, SVM, *k*-nearest neighbors (*k*-NN), and ridge regression. These models were combined via a penalized linear meta-model that assigned the greatest weights to RF and XGBoost, with a modest contribution from ridge regression. Compared with a reported stepwise regression (*R*^2^ = 0.522), the ensemble approach demonstrated superior performance, explaining a significantly greater proportion of the variance in rectal temperature (*R*^2^ = 0.62) (11). This illustrated the advantage of combining complementary models. However, the independent test set results revealed that the improvement observed during validation translated into a modest but consistent advantage for the stacked ensemble, which slightly outperformed RF while maintaining closely comparable predictive behavior. Together, these results suggest that RF is a very strong and relatively parsimonious model for this application, whereas the stacked ensemble provides comparable performance with greater algorithmic complexity.

Two recent ML/IRT studies in dairy cows provide useful comparisons. A stacked AutoGluon ensemble combining medial canthus IRT with respiration rate and temperature-humidity index was used to predict rectal temperature in 295 dairy cows achieving *R*^2^ of 0.73 (35). Another study using optimised XGBoost applied to 3,005 records from dairy cows using trunk IRT, environmental and behavioral variables reported a test set *R*^2^ of 0.540 with MAE of 0.232, comparable to the current study despite the much larger dataset and substantially different feature set (36). The negative result, in which a consumer-grade FLIR One Pro failed to track rectal temperature in 318 dairy calves, is best explained by sensor resolution and ROI placement rather than by any failure of the underlying physiology, an interpretation supported by recent reviews emphasising importance of instrument grade and standardised protocols (37, 38). For buffalo specifically, it has been concluded that thermal-window data in river buffalo were sparse and inconsistent between regions; the present findings begin to address that gap at the medial canthus (39).

The ablation study revealed a clear hierarchy of predictive contribution. Left eye temperature alone explained only 36.6% of test-set variance, rising to 56% when environmental variables were added, and reaching 61.5% with the complete feature set. This progressive improvement confirms that environmental calibration variables contribute meaningfully beyond the ocular signal alone. Notably, the LT with environment model approached the complete feature model’s performance, suggesting that in scenarios where only one eye can be reliably imaged, this approach offers a practical and accurate alternative. Residual analysis confirmed the robustness and reliability of the stacked ensemble model, supporting its application in non-invasive health monitoring of buffaloes.

The stronger predictive value of left eye temperature observed in this study may be related to physiological asymmetry, although this would require a separate experiment to confirm. The right hemisphere of the brain, which controls the left side of the body, plays a dominant role in thermoregulation, resulting in higher temperatures in the left eye (40, 41). This physiological asymmetry has been observed in cattle, where exposure to sunlight resulted in higher left eye temperatures than right eye temperatures (38.8 °C vs. 38.2 °C) (42). This phenomenon may be attributed to differential hemispheric activity and the physiological response to sunlight exposure (43). Earlier studies did not account for non-linear relationships in their analysis, an important consideration in biological systems, and did not find significant differences between left and right eye temperatures via thermal imaging (11, 21).

Repeated observations of a selected group of animals revealed a strong correlation between rectal and IRT-based temperatures, particularly in the left eye. The Bland–Altman analysis revealed a consistent positive bias and narrow limits. Core body temperature changes were reflected in both infrared camera-derived eye temperatures and rectal temperatures, although rectal temperatures were consistently higher by 1.5 °C than left eye readings were. Thermal eye measurements from the left eye showed a strong relationship (*r* = 0.73) with rectal temperature during continuous monitoring, suggesting that left eye temperature measurements can be used to monitor trends in inner body temperatures.

The ensemble model exhibited robust predictive performance when validated against this independent dataset comprising measurements taken at multiple distinct time points from a separate cohort of animals, providing evidence of the model’s reliability, transferability, and capacity to generalize effectively beyond the specific conditions represented in the original training and test datasets. However, the slightly reduced accuracy in the form of the Pearson correlation coefficient compared with that of the main dataset suggests that temporal factors may influence the prediction accuracy. Specifically, the time of day when recordings are obtained appears to be an important variable that should be systematically incorporated into machine learning models designed for continuous monitoring applications. This finding highlights the importance of considering circadian influences when developing robust predictive models for physiological parameters.

The linear mixed-effects model quantifies the statistical association between rectal temperature and the candidate predictors (LR, RT, AT, RH, RefT and time of day) under a repeated-measures design. The ocular thermal temperature at the medial canthus served as a peripheral indicator associated with rectal temperature. The statistically significant effect of time indicated that rectal temperature was dynamic over the day, reflecting diurnal variation (44, 45).

Several limitations should be considered when interpreting these results. First, the study included a relatively small sample size, potentially limiting broader applicability. Furthermore, observations from our new independent continuous monitoring dataset suggest that measurement timing significantly influences rectal temperature prediction accuracy—a variable not captured in our first dataset. Also, this continuous monitoring cohort comprised younger animals (2.5–3.5 years) compared to primary dataset (4–8 years), which may have introduced physiological variability. In the present study, all the animals were adult female, lactating Murrah buffaloes housed at a single research farm under similar management and climatic conditions. This choice was made to provide a focused first evaluation of the ocular IRT and machine learning framework in a relatively homogenous group. Future efforts should extend this framework across breeds, age groups, physiological states, and environmental conditions and assess its sensitivity under hyperthermic states using larger datasets.

Overall, the proposed framework offers a practical, cost-effective and remotely deployable solution for routine temperature surveillance in buffalo herds, reducing handler workload and animal stress in alignment with ethical and sustainable livestock management principles (1, 2, 5, 46). The scalability of ensemble models to other livestock species, including cattle and goats, presents an exciting opportunity for broader animal farming applications. Integrating predictive models into real-time health monitoring systems could facilitate continuous surveillance under diverse environmental conditions, enabling early disease detection and timely intervention, which are critical for precision livestock farming data-driven optimization (47).

## 5 Conclusion

This study shows that infrared thermography of the medial canthus of the eye, combined with ensemble machine learning, can predict rectal temperature in adult lactating Murrah buffaloes with clinically acceptable precision: 96.8% of stacked ensemble predictions on the independent test set fell within ±0.5 °C of the rectal reference. Bootstrap confidence intervals confirmed that random forest and the stacked ensemble (combining RF, XGBoost, support vector machine, *k*-nearest neighbors, and ridge regression via a penalized linear meta-learner) delivered statistically comparable performance at this sample size with RF emerging as the strongest single learner. A systematic ablation study highlighted the distinct contribution of each feature group: left medial canthus temperature alone explained 36.6% of test-set variance, rising to 56.0% with environmental variables and 61.5% with full feature set. The 48-h repeated measures experiment further demonstrated that eye temperature tracks rectal temperature over time with consistent mean offset of about 1.5 °C. Taken together, these findings support ocular medial canthus thermography combined with ensemble machine learning as a welfare-friendly, contact-free alternative to rectal thermometry for routine temperature surveillance in adult Murrah buffaloes.

## Data Availability

The raw data supporting the conclusions of this article will be made available by the authors, without undue reservation.
