# Supplementary material for: Ocular infrared thermography combined with stacked ensemble machine learning enables contact-free rectal temperature prediction in Murrah buffaloes
Source: Front Vet Sci. 2026 Jul 28;13:1859307. doi: 10.3389/fvets.2026.1859307 (PMC13457474; doi:10.3389/fvets.2026.1859307)
Supplement: Supplementary file 1 [file Image_1.pdf]

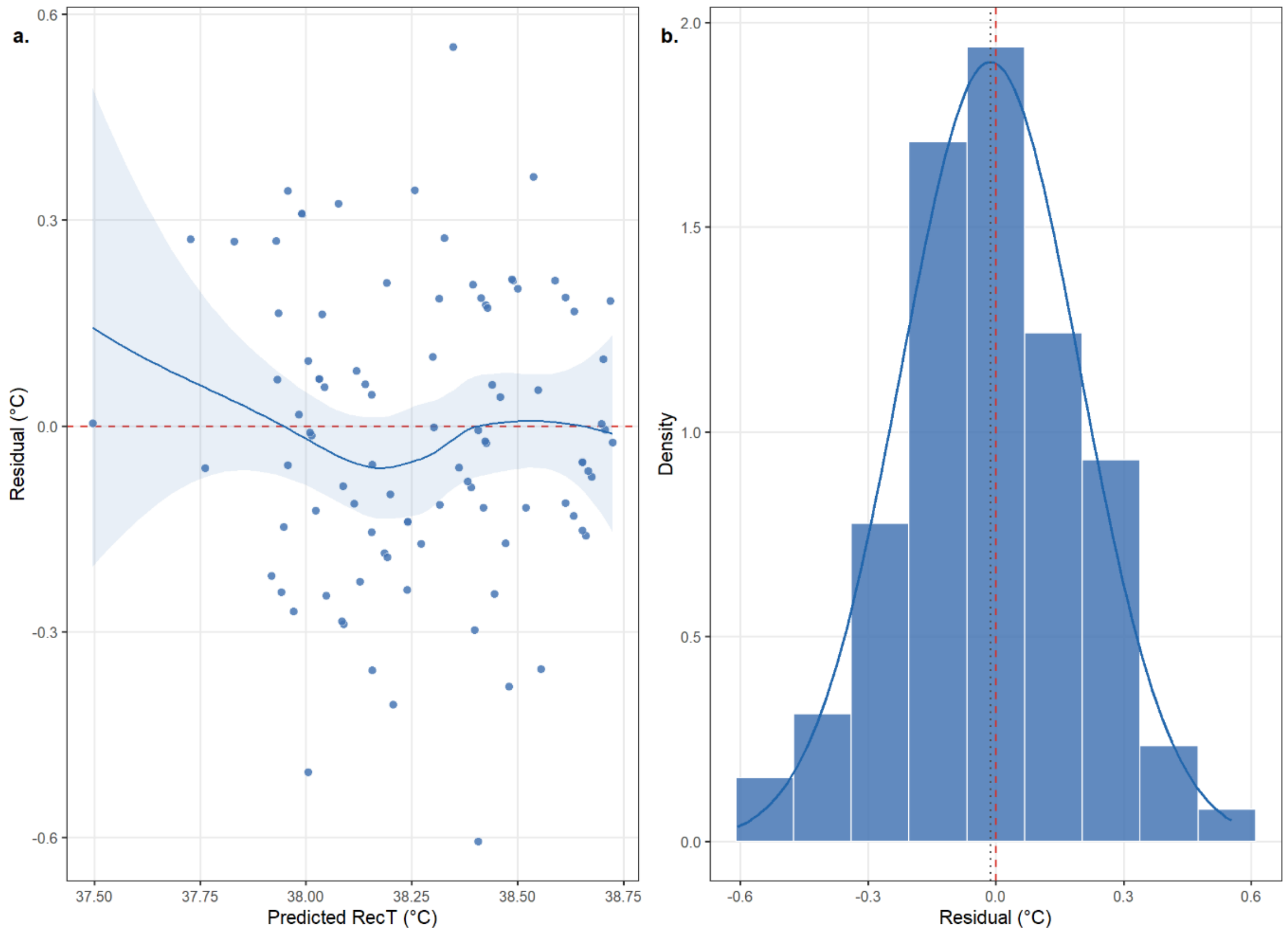

Supplementary Figure S1: Residual analysis of the stacked ensemble on the independent test set. (a) Residuals plotted against predicted rectal temperature; red dashed line marks zero and the blue band shows the LOESS trend with 95% confidence interval. (b) Distribution of residuals overlaid with a normal density curve; red dashed line marks zero and grey dotted line marks the mean residual.

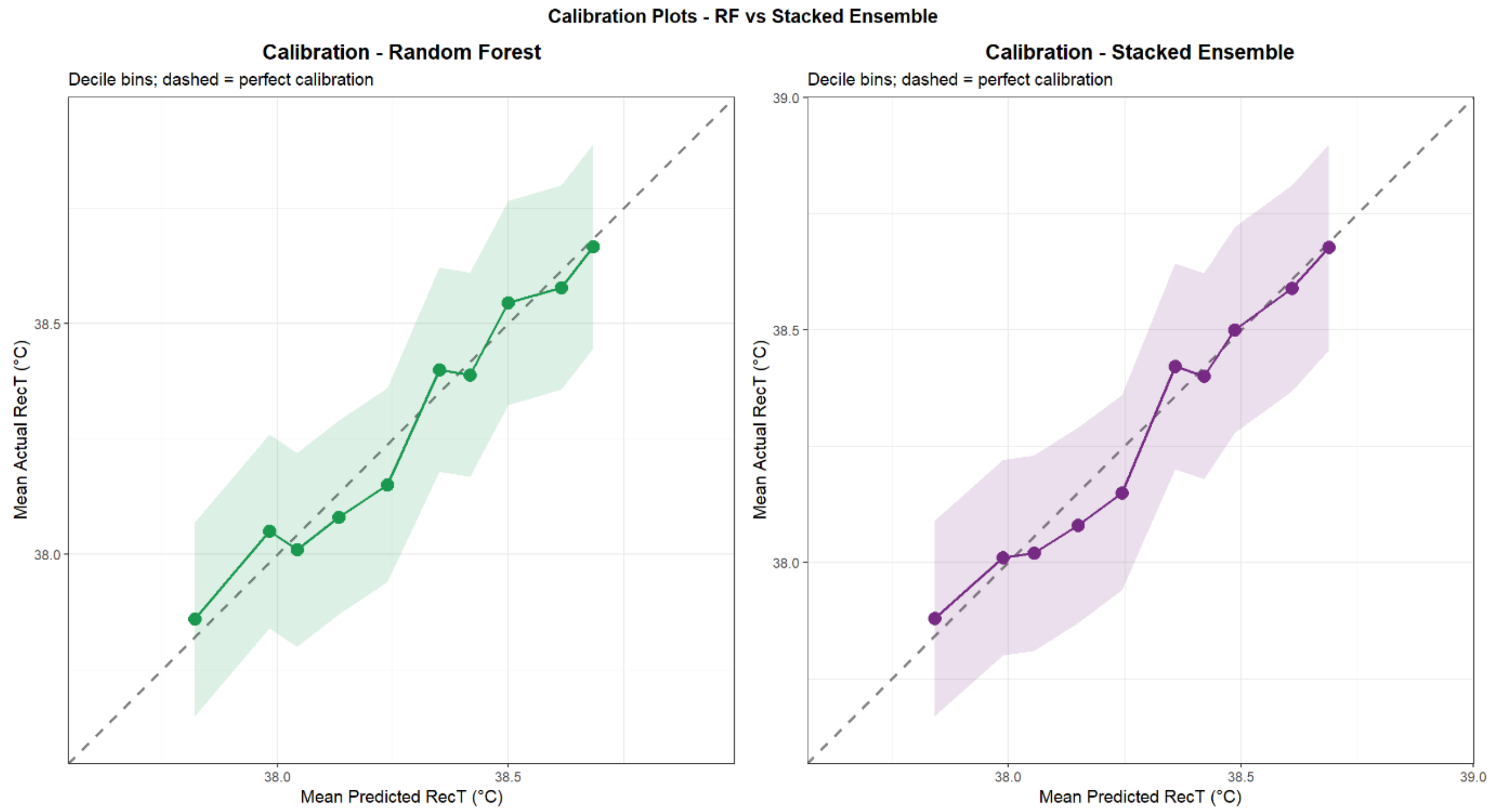

Supplementary Figure S2: Calibration plots for the random forest (RF) and stacked ensemble models on the independent test set (n=95). The dashed line represents perfect calibration (predicted = observed). Both models show good calibration across the observed temperature range, with no evidence of systematic over- or underprediction at either extreme.
